# Effect and mechanism of resveratrol on ferroptosis mediated by p53/SLC7A11 in oral squamous cell carcinoma

**DOI:** 10.1186/s12903-024-04395-3

**Published:** 2024-07-10

**Authors:** Chen Mao, Liqiang Gong, Wenming Kang

**Affiliations:** Department of Stomatology, Loudi Central Hospital of Hunan Province, 51 Changqing Middle Street, Loudi, 417000 Hunan China

**Keywords:** Resveratrol, Oral squamous cell carcinoma, Ferroptosis, p53, SLC7A11, Cell malignant behaviors

## Abstract

**Objective:**

Resveratrol (Res) is a natural phytoestrogen with antitumor activity. This study sought to investigate the role of Res in ferroptosis in oral squamous cell carcinoma (OSCC).

**Methods:**

Normal human oral keratinocyte (HOK)/oral OSCC (CAL-27/SCC-9) cell lines were treated with different doses of Res. Res toxicity was determined by MTT assay, with half maximal inhibitory concentration values of Res on CAL-27 and SCC-9 cells calculated. Cell viability/colony formation efficiency/migration/invasion/cycle were assessed by CCK-8/colony formation assay/transwell assay/flow cytometry. The expression of p53 protein in the nucleus and cytoplasm, glutathione peroxidase 4 (GPX4) expression, and SLC7A11 messenger RNA (mRNA) and protein expression levels were determined by Western blot and RT-qPCR. Fe^2+^ content, reactive oxygen species (ROS) level, reduced glutathione (GSH), and lactate dehydrogenase (LDH) release were assessed.

**Results:**

Medium- to low-dose Res had no toxic effect on HOK cells, while high-dose Res markedly reduced HOK cell viability. Res significantly suppressed the viability of OSCC cells (CAL-27 and SCC-9). Res inhibited OSCC cell colony formation/migration/invasion, and induced G1 phase arrest. Res caused the translocation of p53 protein to the nucleus, obviously increased Fe^2+^ content, ROS level and LDH release, decreased GSH content and GPX4 protein expression, and induced ferroptosis. Down-regulation of p53 partially reversed the inhibitory effects of Res on CAL-27 cell malignant behaviors. Res inhibited SLC7A11 transcription by promoting p53 entry into the nucleus. SLC7A11 overexpression negated the the regulatory effects of p53 knockout on the role of Res in OSCC cell malignant behaviors and ferroptosis.

**Conclusion:**

Res accelerated ferroptosis and inhibited malignant behaviors in OSCC cells by regulating p53/SLC7A11.

**Supplementary Information:**

The online version contains supplementary material available at 10.1186/s12903-024-04395-3.

## Introduction

Oral squamous cell carcinoma (OSCC) is an invasive and metastatic oral malignancy with growing incidences [1, 2]. The excision of the primary tumor, with or without lymph node dissection, is a routine treatment for OSCC [3]. Due to the late clinical discovery, high incidence of local infiltration and metastasis, and poor prognosis, the five-year survival rate of OSCC has not improved significantly in recent years despite advances in therapeutic strategies [4]. In addition, the molecular mechanisms and genetic basis of OSCC tumorigenesis have remained a mystery so far [5]. Accordingly, it is of great significance to grasp the precise molecular mechanism of OSCC tumorigenesis and develop new therapeutic methods.

Ferroptosis is a new form of regulated cell death that is morphological, biochemical, and genetically distinct from autophagy and apoptosis and is characterized by iron-dependent reactive oxygen species (ROS) accumulation and irresistible lipid metabolism [6, 7]. As reported, ferroptosis is closely associated with many diseases, such as cancer, neurological diseases, and kidney damage [8], and thus it is perceived as a potential prevention strategy to trigger cancer cell death, especially in malignant tumors that are resistant to conventional therapies [9]. A previous study has verified that ferroptosis plays an essential role in OSCC [10]. Besides, some genes that promote the proliferation of OSCC cells, such as GPX4 and SREBP, seem to protect cells from ferroptosis [11]. Recently, solute carrier family 7 member 11 (SLC7A11) has been verified to be closely negatively correlated with ferroptosis, and SLC7A11 inhibition can induce ferroptosis [12, 13]. Zhu et al. have reported upregulated SLC7A11 in OSCC [14]. To date, however, the specific mechanism of ferroptosis in OSCC has not been properly portrayed.

Resveratrol (Res) is a plant antitoxin with distinct bioactive polyphenols that can prevent several human diseases, including cancer [15, 16]. Recent research has indicated that Res exhibits anti-cancer properties in colorectal cancer (CRC) and lung squamous cell carcinoma via its ability to induce ferroptosis [17, 18]. Meanwhile, Res, as an important component of red wine, has antioxidant, anti-tumor, and anti-inflammatory properties [19, 20]. Res has been generally concerned since it can inhibit the proliferation of tumor cells, provoke apoptosis, or sensitize tumor cells to the effects of radiotherapy and chemotherapy [21–23]. Numerous studies have revealed the modulation of Res in intracellular molecular processes involved in cell apoptosis in diverse cancer types, such as head and neck squamous cell carcinoma (HNSCC) [24–26]. Intriguingly, Res can lower the risk of OSCC and other cancers [27]. Nevertheless, reports on the role of Res in OSCC by governing ferroptosis are insufficient.

p53, as a transcription factor, principally coordinates its expression by binding to p53 DNA-binding elements in target genes to achieve tumor-suppressive function [28]. It has been identified that p53 inhibits cystine uptake and sensitizes cells to ferroptosis by inhibiting the expression of SLC7A11 [29]. Moreover, Res promotes cell cycle arrest at the G1 phase in OSCC cells, thereby regulating the expression levels of cell cycle-related proteins and inhibiting cell proliferation [30, 31]. Cyclin D and cyclin E are major regulators of cell cycle progression and are indispensable for G1 phase progression [32]. Besides, the involvement of p53-mediated cell cycle arrest is also crucially critical in the progression of cancer [29]. However, there are no reports that Res mediates G1 phase arrest of the cell cycle and promotes ferroptosis by regulating p53/SLC7A11 in OSCC. This study aims to elucidate the specific mechanism of Res in coordinating ferroptosis in OSCC cells.

## Materials and methods

### Cell culture

Normal human oral keratinocyte cell lines (HOK) and OSCC cell lines CAL-27/SCC-9 were obtained from ATCC (Manassas, VA, USA). HOK cells were cultured in Dulbecco’s modified Eagle medium (DMEM) (Cat#11,995,500 TB; Gibco, Carlsbad, CA, USA), whereas CAL-27 and SCC-9 cells were cultured in DMEM/F12 (Cat#C11330500BT; Gibco) supplemented with 10% fetal bovine serum (FBS) (Cat#10,099,141 C; Gibco), as well as 100 U/mL of streptomycin and penicillin (Gibco) [33]. Cells were cultured in a humidified incubator containing 5% CO_2_ at 37 °C.

### Reagent

Res was acquired from Sigma (St. Louis, MO, USA). Apoptosis inhibitor ZVAD-FMK (No. S7023) and ferroptosis inhibitor ferrostatin-1 (No. S7243) were obtained from Selleck (Houston, TX, USA), and cell necrosis inhibitor necrosulfonamide (ab143839) was obtained from Abcam (Cambridge, MA, USA). The P53 inhibitor pifithrin-α (PFT-α) was procured from Topscience (Shanghai, China).

### Cell transfection and grouping

The SLC7A11 small interfering vector (si-SLC7A11) and matched control (si-NC) synthesized by Ribobio (Guangzhou, China) were transfected into CAL-27 cells utilizing the Lipofectamine 2000 (Promega, WI, USA).

Cells were grouped as below: HOK (normally cultured HOK without any treatment), Blank (normally cultured CAL-27/SCC-9 cells without any treatment), Res group (CAL-27/SCC-9 cells were cultured with 50 µM Res for 48 h), Res + ferrostatin-1 group (CAL-27/SCC-9 cells were cultured with 50 µM Res and 500 nM ferrostatin-1 for 48 h [34]), Res + ZVAD-FMK group (CAL-27/SCC-9 cells were cultured with 50 µM Res and 20 µM ZVAD-FMK for 48 h [34]), Res + necrosulfonamide group (CAL-27/SCC-9 cells were cultured with 50 µM Res and 1 µM necrosulfonamide for 48 h [34]), Res + dimethyl sulfoxide (DMSO) or Res + PFT-α group (CAL-27/SCC-9 cells were cultured with 50 µM Res and 5 µM PFT-α or the same amount of DMSO for 48 h [35]), Res + PFT-α + si-NC or Res + PFT-α + si-SLC7A11 group (CAL-27 cells transfected with si-NC or si-SLC7A11 were cultured with 50 µM Res and 5 µM PFT-α for 48 h).

### Toxicity determination and half maximal inhibitory concentration (IC50) value calculation

HOK cells and OSCC cells (CAL-27 and SCC-9) were treated with different doses of Res (10, 25, 50, 75, and 100 µM) by the 3-(4,5-dimethyl-2-thiazolyl)-2,5-diphenyl-2-H-tetrazolium bromide (MTT) method. The toxicity was determined, and the IC50 values of Res on CAL-27 and SCC-9 cells were calculated. Put simply, cells (1 × 10^5^/well) were seeded on to 96-well plates and cultured for 24 h. After treatment as per the above groups, cells were added with MTT solution with a final concentration of 0.5 mg/mL at 37 °C for 4 h, and then added with 100 µL of DMSO in each well. The optical density (OD) value was measured using a microplate reader (Thermo Fisher Scientific, Waltham, MA, USA) at a wavelength of 450 nm. The calculation of the cell viability ratio was performed using the following formula: cell viability ratio (%) = (A_sample_ - A_blank_)/(A_control_ - A_blank_) × 100 [35].

### Cell counting kit-8 (CCK-8) assay

Cell viability was examined using the CCK-8 kits (Seyotin, Guangzhou, China). The prepared cell suspension (1 × 10^5^ cells, 100 µL) was supplemented to 96-well plates and cultured for 12, 24, and 48 h, respectively. Following the group treatment described above, a 10 µL solution of CCK-8 was introduced into each well and the samples were cultured at room temperature for 4 h, followed by OD measurement at 450 nm and cell viability assessment using a microplate reader (Thermo Fisher Scientific). The calculation method for cell viability was consistent with that in the MTT assay.

### Colony formation assay

Cells were seeded in 6-well plates (300 cells/well) and incubated for 2 weeks to form colonies. After incubation, the cells were fixed with formalin and stained with the crystal violet solution to observe plaques. The plaques were observed under a microscope (DM4000B), and quantitative analysis was performed by Image J software (National Institutes of Health, Bethesda, MD, USA).

### Determination of cell migration and invasion

Cell migration assay was carried out in a 24-well Transwell chamber (Corning, NY, USA), which included an 8 μm pore size polycarbonate membrane filter and was precoated with 100 µg Matrigel (Becton-Dickinson, MA, USA) for invasion assay. In short, cells were seeded in the upper chamber and cultured in 500 µL RPMI 1640 medium without FBS, whereas 500 µL medium containing 10% FBS was placed in the lower chamber. The plates were incubated in a humidified incubator for 24 h at 37 °C with 5% CO_2_. Cells on the upper side of the filter were then removed with cotton swabs. Thereafter, the lower cells were fixed with 4% formaldehyde and stained with 1% crystal violet in PBS for 5 min. The cells underneath the filter were defined as migrating cells and counted in five random fields of each filter (× 200 magnification). Image J software (National Institutes of Health) was used for quantitative analysis.

### Evaluation of cell death by lactate dehydrogenase (LDH)

According to the manufacturer’s instructions, the release of LDH was determined using the LDH detection kit (Beyotime, Shanghai, China) to evaluate cell death, and the experiment was repeated three times. The OD value of the sample was measured using a microplate reader (Thermo Fisher Scientific) at 490 nm, and the release of LDH was calculated. LDH release (%) = (OD_experimental group_ - OD_blank control group_)/(OD_control group_ - OD_blank control group_) × 100%.

### Determination of Fe^2+^ content

The iron determination kit (Abcam) was employed to measure Fe^2+^ content. After OSCC cells were collected, 5 µL of detection buffer and 100 µL of iron probe were successively added to the cells, mixed evenly, and then detected at 37 °C. Cells were incubated for 60 min, with the OD value determined at 593 nm.

### Detection of reactive oxygen species (ROS) levels

ROS levels were measured using the ROS detection kit (S0033, Beyotime) in the light of the manufacturer’s manuals. Later, a fluorescence microscope (Leica Microsystems, Wetzlar, Germany) was used for observation and photo shoots.

### Determination of glutathione (GSH)

The GSH levels were measured using the GSH kits (Jiancheng, Nanjing, China). The harvested cells were broken by ultrasound to obtain the supernatant for measuring GSH levels in a fluorescence microplate reader (Varioskan & LUX, Thermo Fisher). The GSH level was expressed as the ratio of the measured absorbance value to that of the control cells at 405 nm.

### Reverse transcription quantitative polymerase chain reaction (RT-qPCR)

Total RNA was isolated utilizing the TRIzol reagent (Thermo Fisher), followed by reverse transcription using the reverse transcription kits (Thermo Fisher). Subsequently, RT-qPCR was performed on the cDNA using the SYBR Green qRT-PCR Mix (Takara, Shiga, Japan) following the manufacturer’s protocol. Using glyceraldehyde-3-phosphate dehydrogenase (GAPDH) as an internal reference, the relative expression of genes was calculated by the 2^−ΔΔCT^ method [36]. Forward and reverse primers are documented as follows: SLC7A11 (F: 5’-ATGCTGAATTGGGAACAACTA-3’ and R: 5’-TTTATAGTTGTTCCCAATTCA-3’); GAPDH (F: 5’-GGAGCGAGATCCCTCCAAAAT-3’ and R: 5’-GGCTGTTGTCATACTTCTCATGG-3’).

### Western blotting

The bicinchoninic acid protein detection kits (Seyotin) were adopted to extract and detect proteins from CAL-27 cells. Protein samples of the same mass were loaded onto 8–12% sodium dodecyl sulfate polyacrylamide gel electrophoresis gel and electrophoretized on ice for 90 min. The isolated proteins were then transferred to polyvinylidene fluoride membranes and blocked with 5% bovine serum albumin for 1 h. The membranes were placed in 5% skim milk prepared with Tris-buffered Saline Tween-20, shaken, and blocked for 1 h, followed by incubation with primary antibodies rabbit anti-p53 (1:2000, ab179477), SLC7A11 (1:1000, ab175186), and GPX4 (1:1000, ab125066) overnight. After washing, horseradish peroxidase-labeled secondary antibody goat anti-rabbit IgG (1:2000, ab6717) was added and incubated for 2 h. Eventually, an enhanced chemiluminescence system (Seyotin) was used for imprinting detection, with GAPDH (1:1000, ab9485) and Lamin B1 (1:1000, ab229025) as internal references. All antibodies were acquired from Abcam.

### Cell cycle detection by flow cytometry

Cells (1 × 10^6^/mL) were fixed overnight with 70% ethanol at 4 °C, stained with propidium iodide (50 µg/mL, Guduo Biotechnology, Shanghai, China) and heated at 37 °C for 30 min. An FACSCaliber flow cytometry (BD Biosciences, San Jose, CA, USA) was used to analyze the number of cells at different stages of the cell cycle and calculate the corresponding percentage.

### Statistical analysis

SPSS21.0 (IBM Corp., Armonk, NY, USA) software and GraphPad Prism 8.0 software (GraphPad Software Inc., San Diego, CA, USA) were used for plotting. Measurement data were expressed in the form of mean ± standard deviation (SD). The *t* test was applied for data comparison between the two groups. One-way analysis of variance (ANOVA) was applied for data comparison among multiple groups, and Tukey’s test was applied for the post-hoc test. The cell experiment was conducted three times. A value of *P* < 0.05 meant statistical significance.

## Results

### Res inhibited the malignant behaviors of OSCC cells

**Fig. 1:**
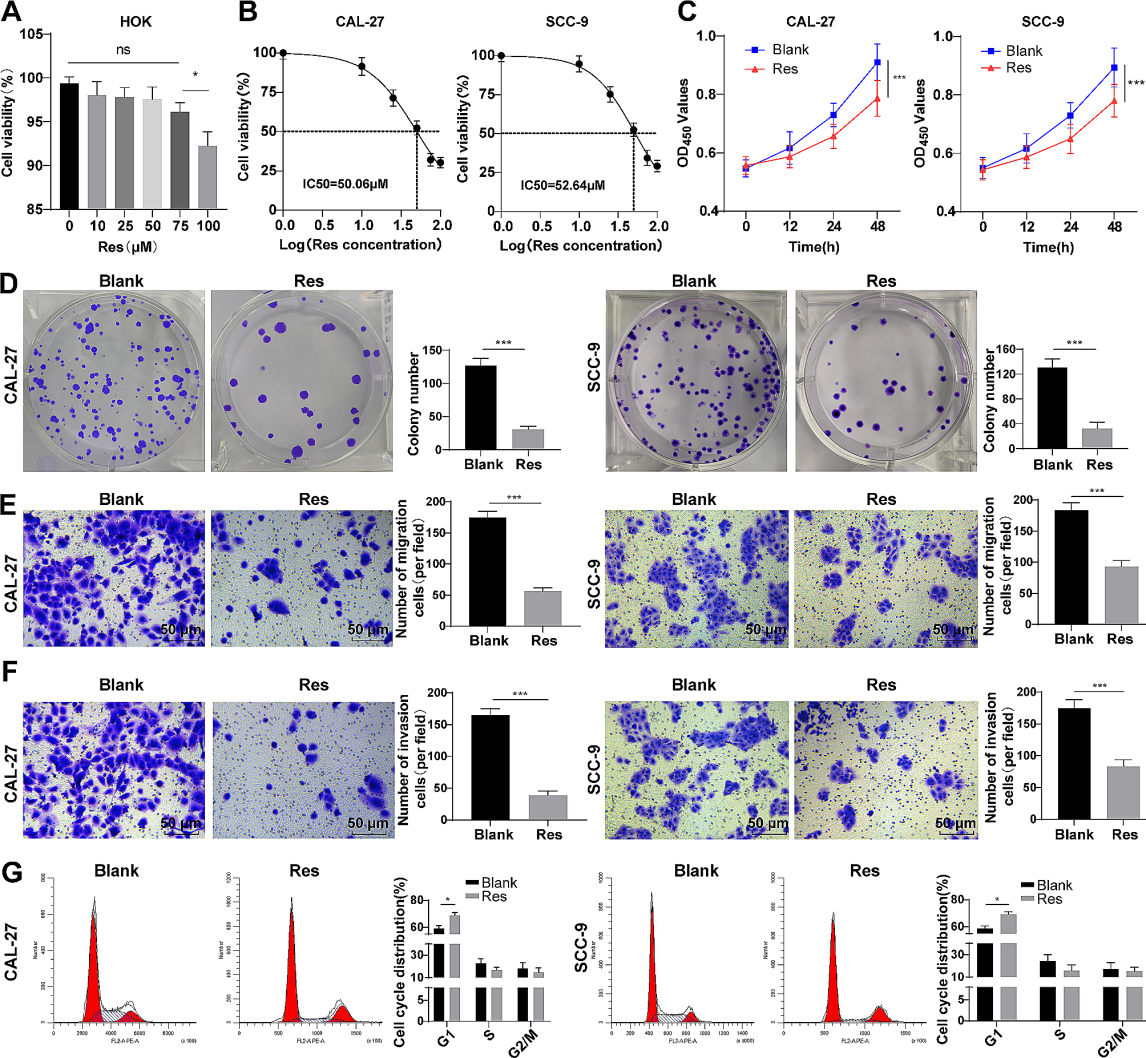
Res inhibited the malignant behaviors of OSCC cells. **A**: The toxicity was determined by MTT; **B**: Cell viability was assessed by MTT assay and IC50 value was calculated; **C**: Cell viability was detected by CCK-8; **D**: Colony formation assay was utilized to test the colony formation efficiency; **E**-**F**: Transwell assay was used to detect cell migration and invasion. **G**: Flow cytometry was utilized to detect the cell cycle (percentage of cells in G1, S, and G2/M phases). The cell experiment was performed three times. Data were expressed as mean ± SD. The *t* test was used for data comparison between the two groups, and Tukey’s test was used for the post-hoc test. ns represented *P* > 0.05, * *P* < 0.05, *** *P* < 0.001

First, HOK and OSCC (SCC-9/CAL-27) cell lines were treated with 10, 25, 50, 75, and 100 µM Res, and the toxicity was assessed by MTT and the IC50 was counted. Based on the results, medium- and low- doses of Res (10, 25, 50, and 75 µM) exhibited no toxic effect on HOK cells (Fig. 1A, all *P* > 0.05), while treatment with high-dose of Res (100 µM) led to noticeably decreased cell viability of HOK cells (Fig. 1A, *P* < 0.05); but the cell viability of both was distinctly inhibited by Res in a concentration-dependent manner after Res treatment. Then, the IC50 value of Res to CAL-27 was calculated to be 50.06 µM by GraphPad Prism 8.0 software, and the IC50 value of Res to SCC-9 was 52.64 µM (Fig. 1B). Consequently, we subsequently treated CAL-27 and SCC-9 cells with 50 µM Res to further explore the anti-cancer effects of Res. CCK-8 and colony formation experiments showed that Res saliently curbed CAL-27 and SCC-9 cell viability and colony formation efficiency compared with the control group (Fig. 1C-D, *P* < 0.001). In addition, Transwell assay illustrated that Res also suppressed the migration and invasion activity of SCC-9/CAL-27 cells (Fig. 1E-F, *P* < 0.001). As reflected by the results of flow cytometry, the percentage of cells in the G1 phase increased clearly after Res treatment of CAL-27 and SCC-9 cells (Fig. 1G, all *P* < 0.001). These results suggested that Res repressed the malignant behaviors of OSCC cells.

### Res induced ferroptosis with up-regulation of p53 in OSCC cells

**Fig. 2:**
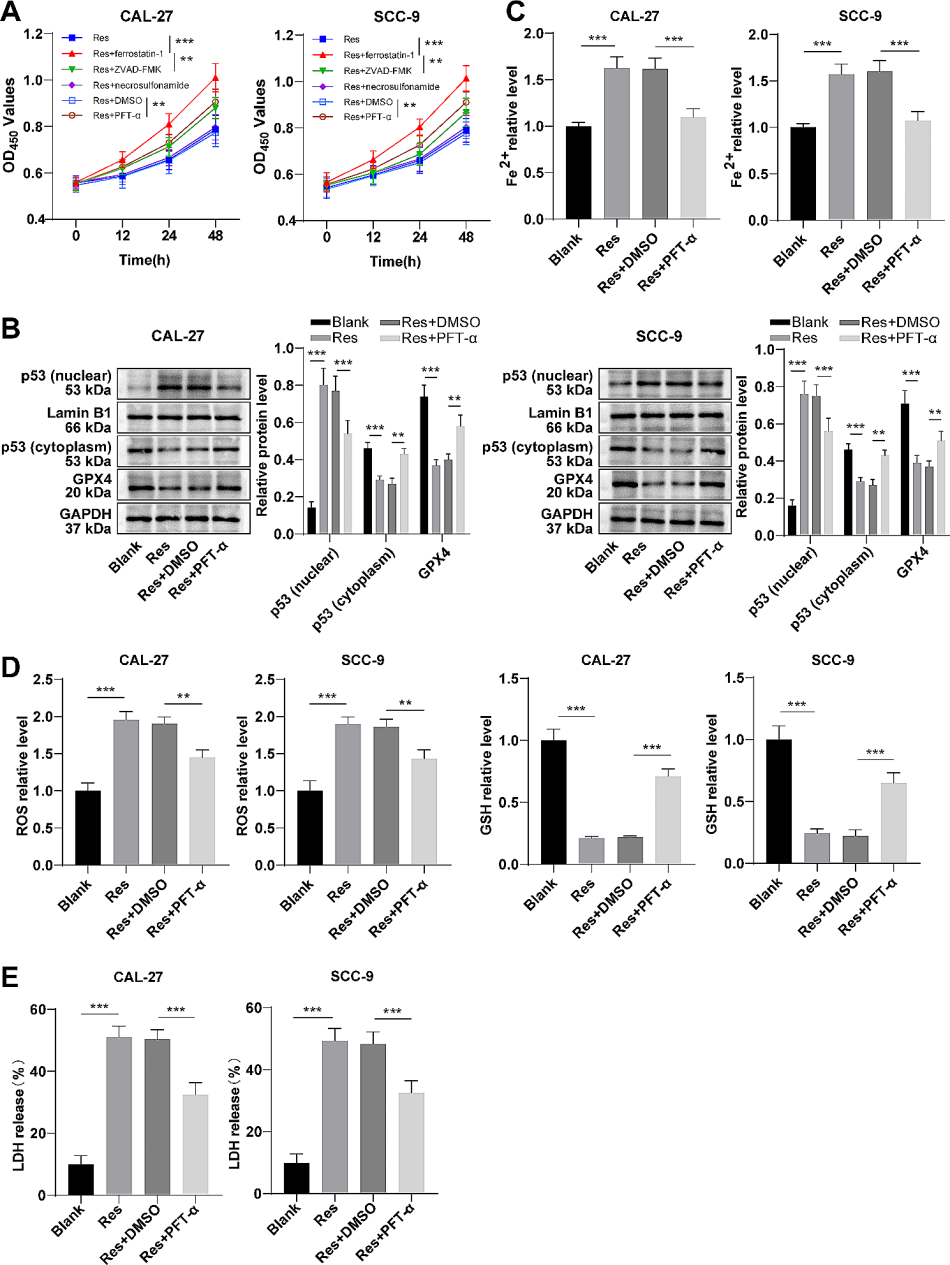
Res induced ferroptosis with up-regulation of p53 in OSCC cells. **A**: Cell viability was evaluated by CCK-8. **B**: p53 expression in the nucleus and cytoplasm and GPX4 protein expression were determined by Western blotting; **C**: The Fe^2+^ content was detected by the kits; **D**: The kits were used to detect the ROS level and GSH content. **E**: LDH release assay was used to evaluate cell death. The cell experiment was performed three times. Data were expressed as mean ± SD. One-way ANOVA was used for data comparison among multiple groups, and Tukey’s test was used for the post-hoc test. ** *P* < 0.01, *** *P* < 0.001

Subsequently, we further investigated the mechanism of Res inhibiting the malignant behaviors of OSCC cells. While CAL-27 and SCC-9 cells were treated with 50 µM Res, cell viability rescue experiments were performed using ferrostatin-1, ZVAD-FMK, and necrosulfonamide, respectively. As presented by CCK-8 assay, the cell viability was obviously higher in the Res + ferrostatin-1 group than the Res group (Fig. 2A, all *P* < 0.05), but was slightly higher in the Res + ZVAD-FMK group than the Res group with no significant difference (Fig. 2A, all *P* > 0.05). In addition, the cell viability of the Res + ferrostatin-1 group was remarkably higher than that of the Res + ZVAD-FMK group (Fig. 2A, all *P* < 0.01). Hence, we hypothesized that Res inhibited cell viability mainly by inducing ferroptosis in OSCC cells. Based on the above results and the fact that p53 plays an important role in ferroptosis of cancer cells [29], we speculated that Res induced ferroptosis of OSCC cells through p53. The results of Western blotting displayed that p53 expression was raised in the nucleus and declined in the cytoplasm following Res treatment (Fig. 2B, all *P* < 0.001). Furthermore, the p53 inhibitor PFT-α was used to treat Res-incubated OSCC cells, and inhibition of p53 expression partially annulled the contributory effect of Res on p53 entry into the nucleus (Fig. 2B, all *P* < 0.01). Thereafter, it was noted that Res could raise the iron ion content, ROS level, and LDH release of OSCC cells and diminish the GSH content and GPX4 protein level (Fig. 2A-E, all *P* < 0.001). In addition to this, PFT-α treatment partly counteracted the changes in cell viability, iron ion content, ROS level, GSH content, LDH release, and GPX4 expression that were treated with Res alone (all *P* < 0.01). Taken together, Res promoted ferroptosis in OSCC cells by up-regulating p53 levels.

### Downregulation of p53 partially reversed the inhibitory effects of res on CAL-27 cell malignant behaviors

**Fig. 3:**
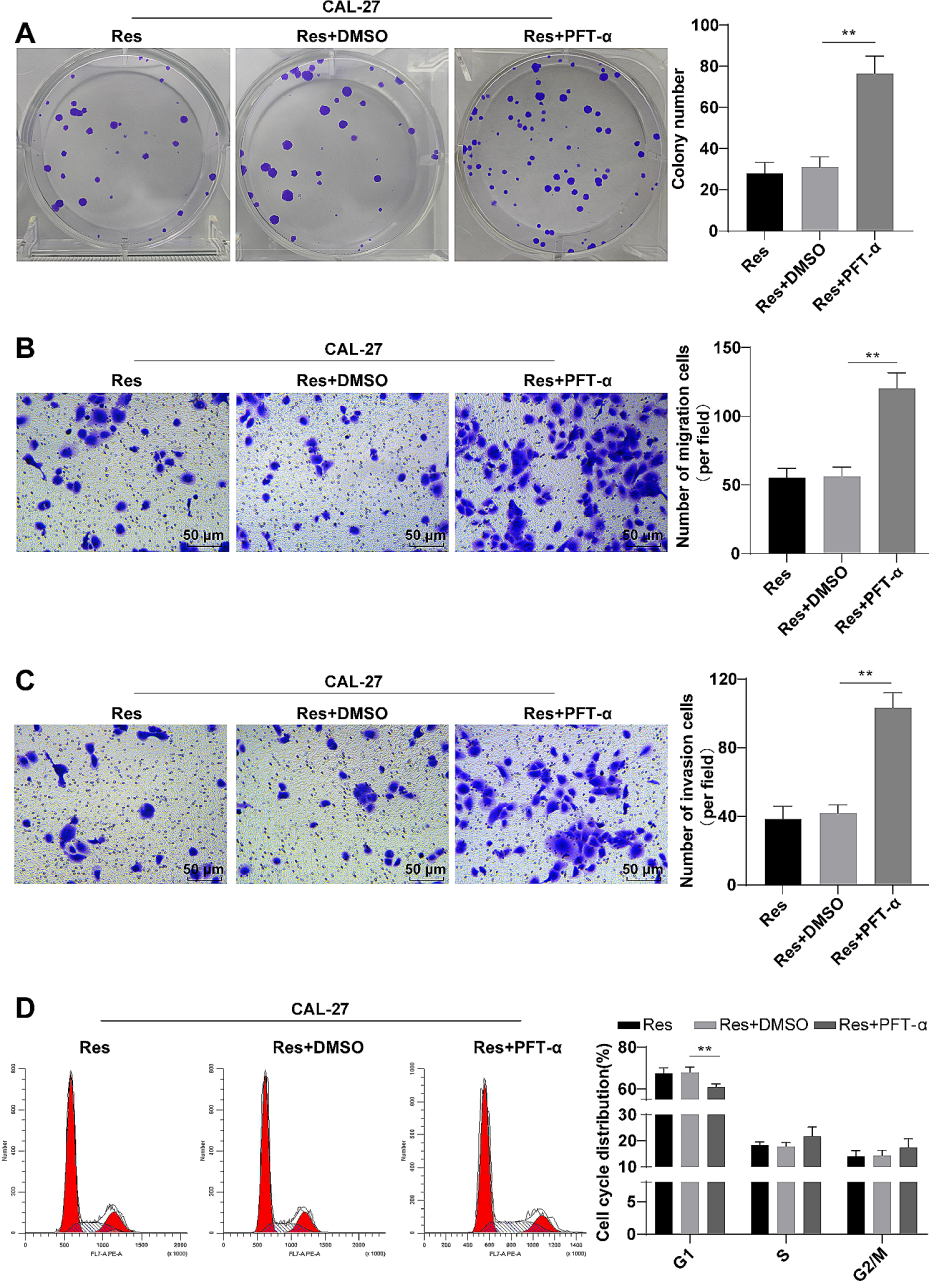
Downregulation of p53 partially reversed the inhibitory effects of Res on CAL-27 cell malignant behaviors. **A**: Colony formation experiment was performed to assess colony formation efficiency; **B**-**C**: Cell migration and invasion abilities were assessed using the Transwell assay; **D**: Cell cycle (percentage of cells in G1, S, and G2/M phases) was detected by flow cytometry. The cell experiment was repeated three times, and the data were expressed as mean ± standard deviation. One-way ANOVA was used for comparisons between multiple sets of data, followed by Tukey’s multiple comparisons test. ** *P* < 0.01, *** *P* < 0.001

To investigate the impact of p53 downregulation on the malignant behaviors of OSCC cells, we chose to utilize CAL-27 cells for future experimental analysis. As detected by colony formation, Transwell and flow cytometry assays, compared to the Res + DMSO group, the Res + PFT-α group showed appreciably elevated colony formation efficiency, migration, and invasion (Fig. 3A-C, all *P* < 0.01), clearly diminished cell percentage in the G1 phase (Fig. 3D, *P* < 0.01). Altogether, knockdown of p53 partially abolished the inhibitory effects of Res on the malignant behaviors of CAL-27 cells.

### Res inhibited SLC7A11 transcription by promoting the entry of p53 into the nucleus

**Fig. 4:**
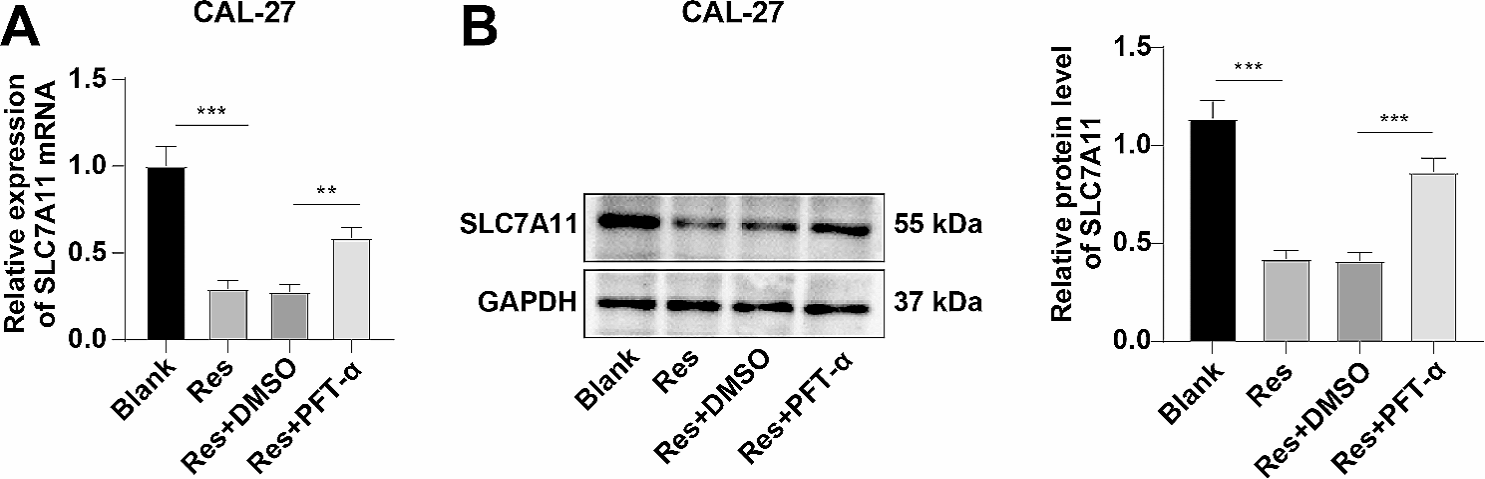
Res inhibited SLC7A11 transcription by promoting the entry of p53 into the nucleus. **A**: The mRNA expression of SLC7A11 was detected by RT-qPCR; **B**: The protein level of SLC7A11 was detected by Western blotting. The cell experiment was performed three times. Data were expressed as mean ± SD. The *t* test was used for data comparison between the two groups, one-way ANOVA was used for data comparison among multiple groups, and Tukey’s test was used for the post-hoc test. ** *P* < 0.01, *** *P* < 0.001

p53, as a tumor suppressor, participates in the regulation of downstream gene transcription through nuclear translocation [37]. Meanwhile, it has been reported that p53 can affect ferroptosis in osteosarcoma by regulating SLC7A11 [35]. Therefore, we hypothesized that Res could affect ferroptosis in OSCC cells by affecting p53 nuclear translocation and regulating the transcription of SLC7A11. As confirmed by RT-qPCR and Western blotting, Res substantially down-regulated the expression of SLC7A11 in CAL-27 cells (Fig. 4A-B, all *P* < 0.001), and the mRNA and protein levels of SLC7A11 were significantly up-regulated in the Res + PFT-α group compared with the Res + DMSO group (Fig. 4A-B, all *P* < 0.01). As a whole, Res limited the transcription of SLC7A11 by facilitating p53 entry into the nucleus.

### Inhibition of SLC7A11 partly annulled the regulatory effect of p53 silencing on the role of res in CAL-27 cell malignant behaviors and ferroptosis

**Fig. 5:**
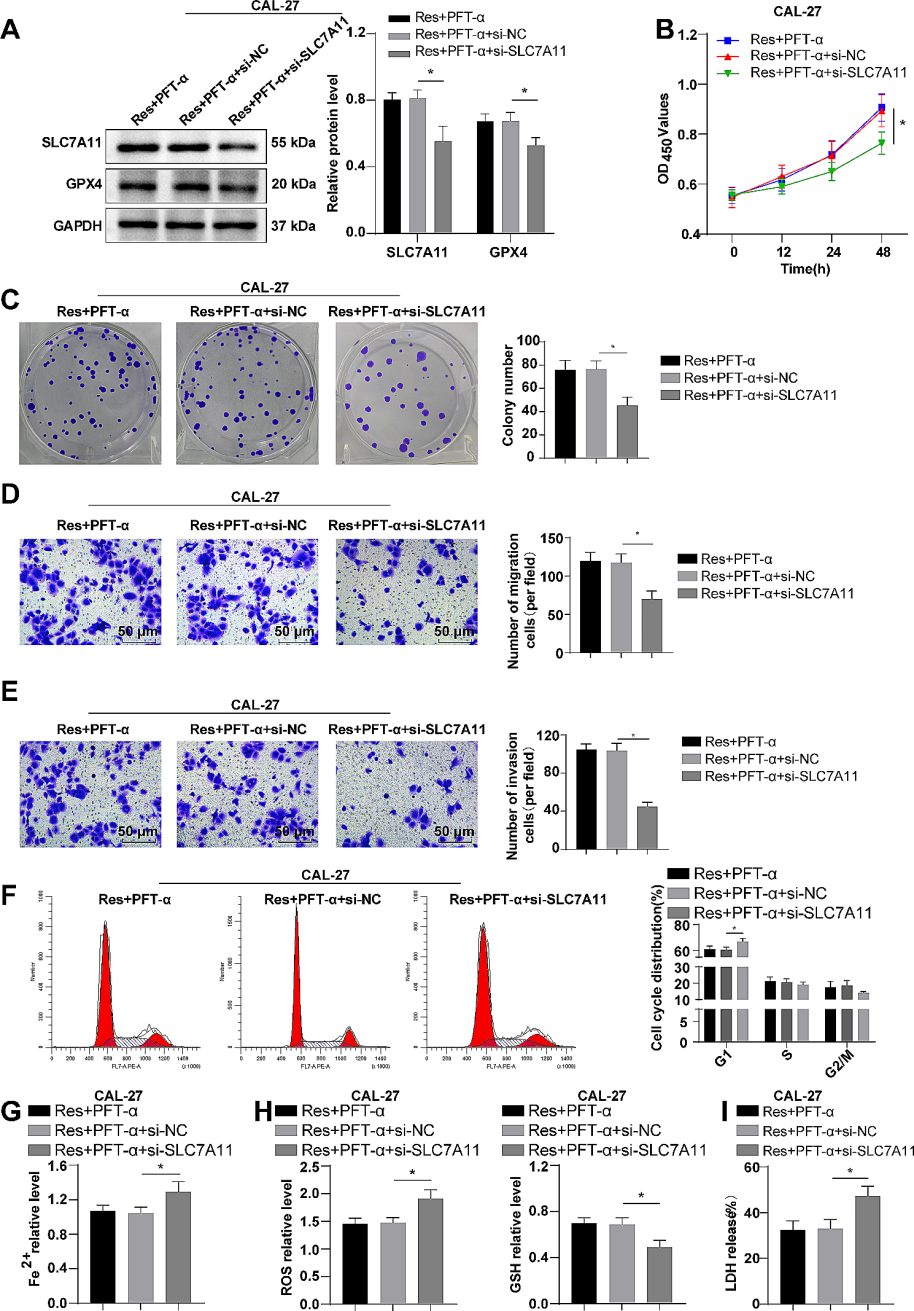
Inhibition of SLC7A11 partly annulled the regulatory effects of p53 silencing on the role of Res in CAL-27 cell malignant behaviors and ferroptosis. **A**: SLC7A11 and GPX4 protein levels were measured by Western blotting; **B**: CCK-8 was used to assess cell viability; **C**: Colony formation experiment was conducted to evaulate colony formation efficiency; **D**-**E**: Transwell assay to assess cell migration and invasion abilities; **F**: Flow cytometry was to detect cell cycle (cell percentages in G1 phase, S phase, and G2/M phase); **G**: Fe^2+^ content was assessed by kit; **H**: The level of ROS and the content of reduced GSH were detected using kits; **I**: LDH release assay to assess cell death. The cell experiment was repeated three times, and the data were expressed as mean ± standard deviation. One-way ANOVA was used for comparisons between multiple sets of data, followed by Tukey’s multiple comparisons test. ** *P* < 0.01

To further verify that Res inhibited the transcription of SLC7A11 through p53 nucleation and promoted the ferroptosis of OSCC cells, we transfected CAL-27 cells with si-SLC7A11 and simultaneously added 50 µM Res and 5 µM PFT-α for 48 h. Western blotting results manifested that SLC7A11 was observably down-regulated in the Res + PFT-α + si-SLC7A11 group relative to the Res + PFT-α + si-NC group (Fig. 5A, *P* < 0.05). Furthermore, in contrast to the Res + PFT-α + si-NC group, the cell viability, colony formation efficiency, migration, and invasion ability of the Res + PFT-α + si-SLC7A11 group were apparently suppressed, and the percentage of cells in the G1 phase was dramatically elevated (Fig. 5B-F, all *P* < 0.05). Additionally, compared to the Res + PFT-α + si-NC group, the Fe^2+^ aggregation, ROS level, and LDH release in the Res + PFT-α + si-SLC7A11 group were augmented, while GSH content and GPX4 protein dropped (Fig. 5A/G-I, all *P* < 0.05). Overall, the impacts of p53 knockout on the role of RES in malignant behaviors and ferroptosis of CAL-27 cells were partially nullified by SLC7A11 suppression.

## Discussion

OSCC is a prevalent malignant neoplasm affecting the regions of the head and neck [38]). Approximately 4.0-8.1% of females and 8.0-8.5% of males are susceptible to developing OSCC, a malignancy that is potentially fatal [39]. Ferroptosis, as described by the programmed cell death of iron metabolism and the accumulation of ROS, plays a crucial role in cancer development and has become a target for the treatment of various cancers [40, 41]. Our findings demonstrated that Res facilitated ferroptosis in OSCC cells by regulating p53/SLC7A11 to induce G1-phase cell cycle arrest in CAL-27 cells.

Accumulating evidence supports that Res can restrain proliferation and facilitate apoptosis of various tumor cells [40, 41]. Importantly, Res inhibits OSCC cell invasion and migration by blocking epithelial-mesenchymal transformation-induced transcription factors [19, 42]. In our present study, Res repressed the CAL-27/SCC-9 cell viability and colony formation efficiency, as well as the formation of colonies and the migration and invasion abilities. Besides, Res suppressed the proliferation of human lung cancer cells by activating p53 and up-regulating p21, resulting in a cell cycle arrest in the G0/G1 phase [43]. The proportion of cells in the G1 phase exhibited a notable rise subsequent to a 24-hour exposure to 100 µM REV in T cell lymphoma [44], which is in line with our study. Briefly, Res restricted the malignant behaviors of OSCC cells.

Although the exact chemoprophylaxis mechanisms of Res are not recognized, they may include: regulating the activity of carcinogenic metabolic enzymes, scavenging free radicals, inhibiting cell proliferation, inhibiting cyclooxygenase activity, inducing apoptosis, and inhibiting angiogenesis [45]. There is increasing evidence that ferroptosis is a key mechanism of tumor suppression [46]. Exploring the molecular mechanism of ferroptosis may be one of the ways to identify new therapeutic targets for cancer [47]. Furthermore, the specific way Res inhibited OSCC cell viability was explored using ferroptosis, apoptosis, and necrosis inhibitors to rescue the viability of OSCC cells. Interestingly, OSCC cells supplemented with Res + ferroptosis inhibitors exhibited higher cellular activity than those treated with Res alone or Res + apoptosis inhibitors, while there was no significant difference between cells supplemented with other inhibitors. Recently, Lee J et al. demonstrated that Res can induce SIRT1 pathway activation and stimulate ferroptosis in HNSCC cells [48]. Based on these data, it is reasonable to speculate that Res suppressed cell viability primarily by inducing ferroptosis. In some cancer cells, p53 activation has been illustrated to be required for ferroptosis [29]. p53 activation modulates ferroptosis responses without apparent effects on GPX4 function [49]. As expected, our study revealed that Res up-regulated p53 protein levels in OSCC cells, enhanced Fe^2+^ content, ROS levels, and LDH release, and decreased the levels of GSH and GPX4 protein in OSCC cells, which can be partially reversed by the p53 inhibitor PFT-a. Likewise, Res enhanced the expression of tumor protein p53 in CRC cells [50]. The cellular process of ferroptosis is initiated by the activation of p53, which exerts its influence via modulating the levels of GSH and ROS [29]. Several studies have demonstrated that Res induces apoptosis by upregulating p53 in many cancer cells [51, 52]. Collectively, our findings initially confirmed that Res encouraged ferroptosis in OSCC cells by up-regulating the expression of p53.

p53 is a crucial tumor suppressor, and the intricacies of its role in regulating the malignant behaviors of cancer cells are widely recognized [53, 54]. In our study, we uncovered that OSCC cells treated with Res + p53 inhibitors exhibited augmented cell colony formation efficiency, invasion, and migration ability, and reduced cell percentages at the G1 phase. Similarly, the inhibitory effect of dual antiplatelet treatment on the migration of non-small-cell lung cancer cells was reversed by the administration of a p53 inhibitor [55]. Also, the downregulation of P53 increases the invasive and metastatic capabilities of prostate cancer cells [56]). The reduction of p53 resulted in the manifestation of a more aggressive phenotype in cancer cells, leading to increased cell motility and invasion in human breast cancer MCF-7 cells [57]. Conclusively, Res inhibited the malignant behaviors of CAL-27 cells, and this effect was partially counteracted by down-regulating p53.

p53 is mainly involved in the regulation of downstream gene transcription via nuclear entry expression [37]. SLC7A11, as an essential amino acid transporter that promotes cysteine supply and GSH synthesis, is a core regulator of the cellular ferroptosis defense system and is a promising therapeutic target in cancer therapy [58]. Notably, p53 affects ferroptosis in osteosarcoma by regulating SLC7A11 [35]. In our study, Res treatment increased the p53 level in the nucleus and lowered it in the cytoplasm of OSCC cells, which is consistent with a previous study showing that Res induced nuclear translocation of p53 in vascular smooth muscle cells [59]. Importantly, Res can down-regulate SLC7A11 levels in OSCC cells, while this phenomenon was negated by p53 inhibition. In line with the existing evidence, suppression of p53 in human CRC cells yielded decreased elastin-induced SLC7A11 expression [7]. As a result, Res repressed the transcription of SLC7A11 by promoting the entry of p53 into the nucleus. However, repression of SLC7A11 reduced cell viability, colony formation efficiency, migration, invasion, GSH content, and GPX4 protein and elevated G1 phase cell percentages, Fe^2+^ accumulation, ROS levels, and LDH release in CAL-27 cells. In a similar light, SLC7A11 inhibition lowers cystine intake and GSH production, resulting in ferroptosis and oxidative damage [13]. Shi et al. reported that tirazamine induced ferroptosis in osteosarcoma cells by down-regulating the expressions of SLC7A11 and GPX4 [60]. Bavachin increases p53 expression, down-regulates SLC7A11 levels, and induces ferroptosis in osteosarcoma cells [35]. The inhibition of SLC7A11-AS1 has been observed to effectively impede CRC cell proliferation and migration while concurrently increasing the intracellular amount of ROS [61]. For the first time, our study uncovered that SLC7A11 suppression partly negated the regulatory effects of p53 knockout on the role of Res in the malignant behaviors and ferroptosis of CAL-27 cells.

In conclusion, this study elucidated the effect of Res on ferroptosis in OSCC cells and verified in vitro that Res reinforced ferroptosis in OSCC cells by promoting the entry of p53 into the nucleus and inhibiting the transcription of SLC7A11, which can provide a certain perspective for the treatment of OSCC. However, this study only verified the mechanism of Res inducing G1 phase cell cycle arrest and promoting ferroptosis in CAL-27 cells by regulating p53/SLC7A11 in CAL-27 cells and failed to explore other mechanisms of ferroptosis influenced by p53. In the future, diverse OSCC cell lines are needed to further verify the results of this study, and whether Res can affect ferroptosis in OSCC through other molecular mechanisms remains to be further explored.

### Electronic supplementary material

Below is the link to the electronic supplementary material.

Supplementary Material 1

## Data Availability

All data generated or analysed during this study are included in this article. Further enquiries can be directed to the corresponding author.
