# Supplementary figures and images for: Effect and mechanism of resveratrol on ferroptosis mediated by p53/SLC7A11 in oral squamous cell carcinoma
Source: BMC Oral Health. 2024 Jul 10;24:773. doi: 10.1186/s12903-024-04395-3 (PMC11238462; doi:10.1186/s12903-024-04395-3)

2B(CAL-27)

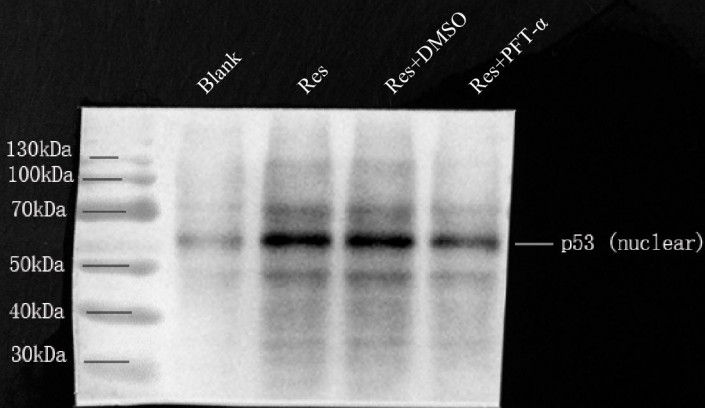

2B(CAL-27)

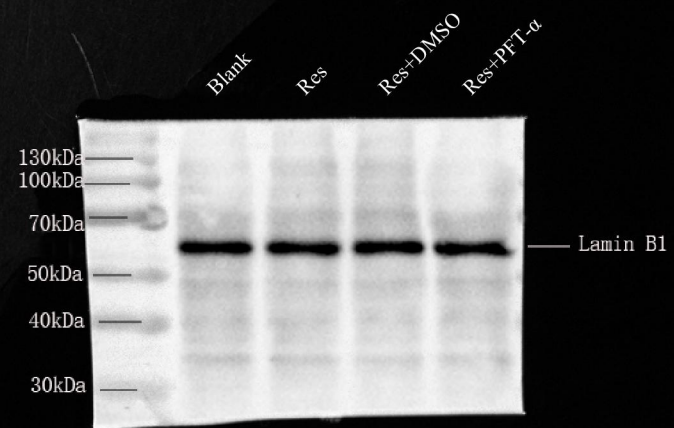

2B(CAL-27)

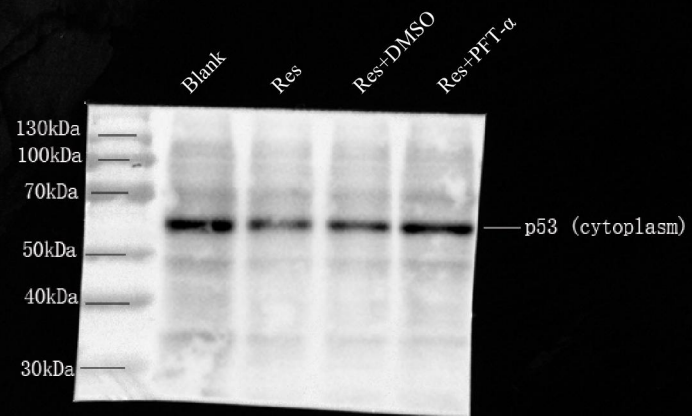

2B(CAL-27)

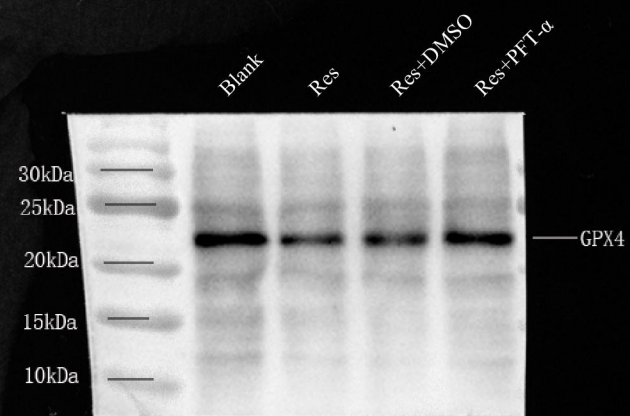

2B(CAL-27)

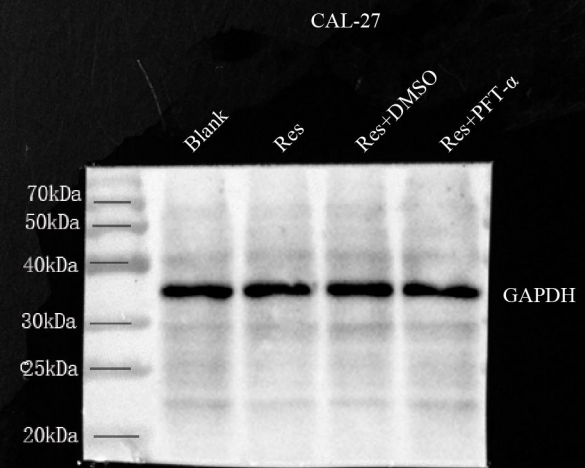

2B(SCC-9)

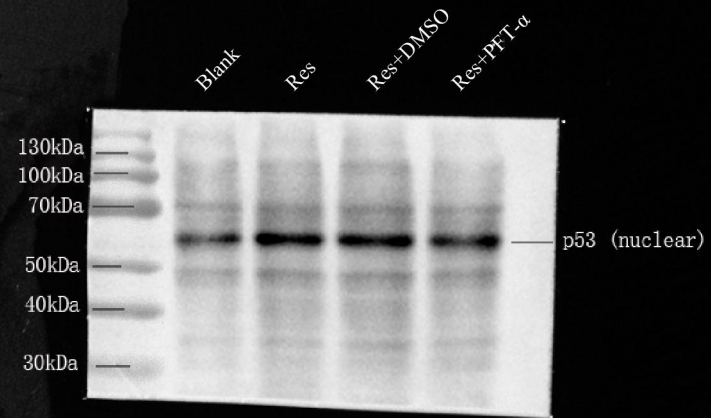

2B(SCC-9)

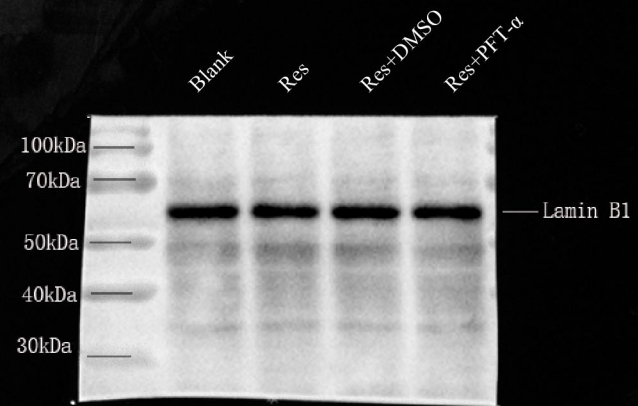

2B(SCC-9)

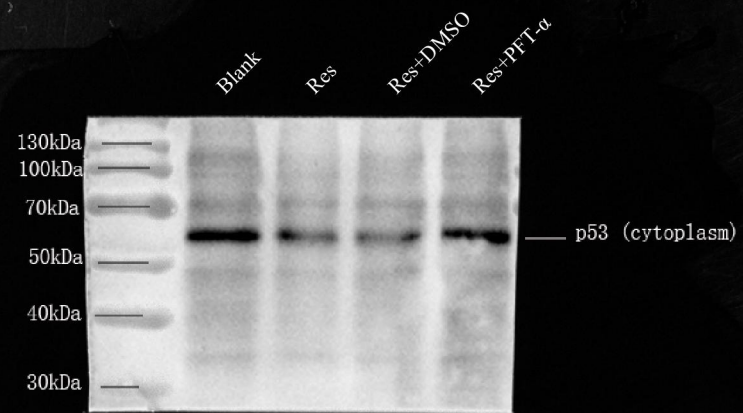

2B(SCC-9)

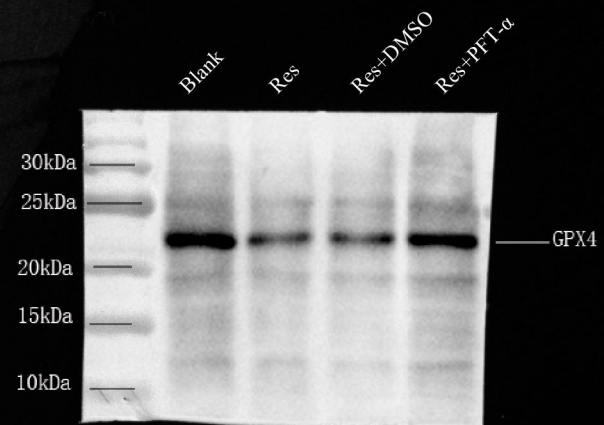

2B(SCC-9)

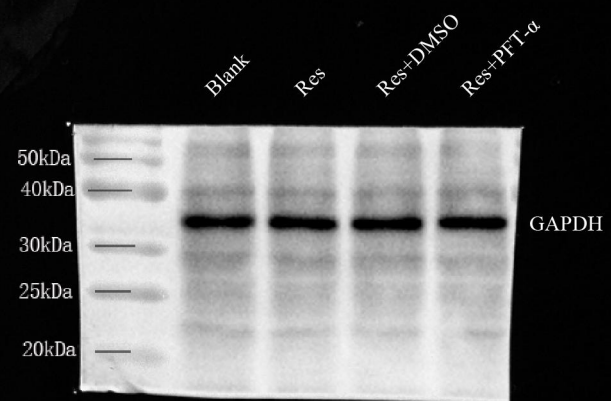

4B

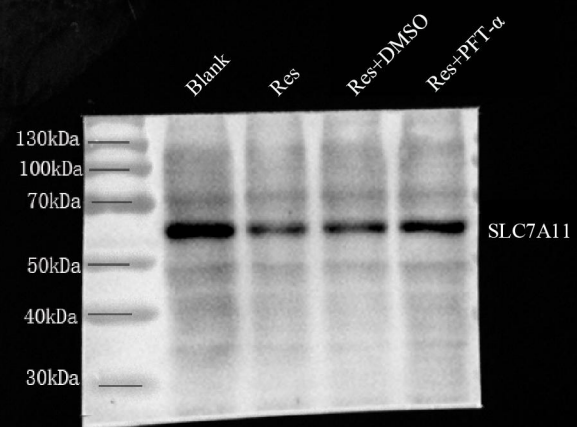

4B

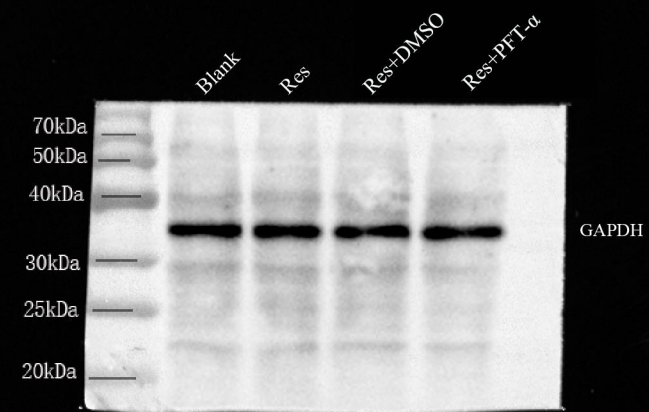

5A(CAL-27)

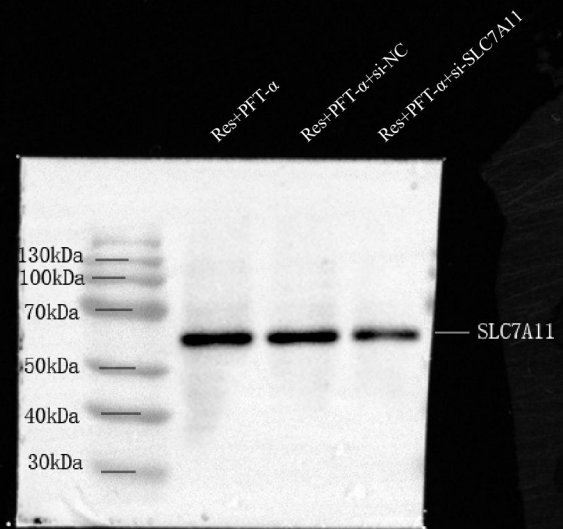

5A(CAL-27)

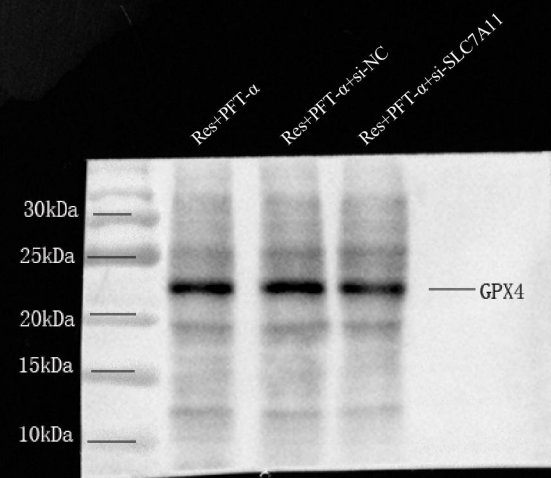

5A(CAL-27)

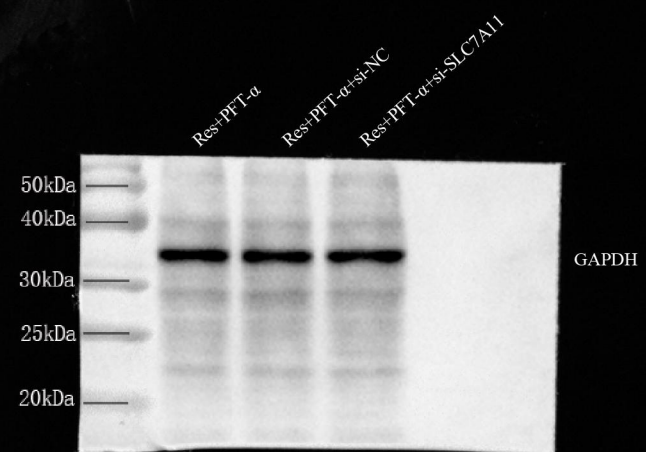

Supplement: Supplementary file 1 — Supplementary Material 1 [file 12903_2024_4395_MOESM1_ESM.pdf]
